# Unmasking an Enigma: The Radiological Spectrum of Disseminated Cysticercosis

**DOI:** 10.7759/cureus.91093

**Published:** 2025-08-27

**Authors:** Keerthi A G, Venkata Sai Pulivadula Mohanarangam

**Affiliations:** 1 Radiology, Sri Ramachandra Institute of Higher Education and Research, Chennai, IND

**Keywords:** cross-sectional imaging, cysticercosis, neurocysticercosis, taenia solium, ultrasonography

## Abstract

Disseminated cysticercosis (DCC) is a rare parasitic infection caused by the systemic spread of *Taenia solium* larvae. This study presents a retrospective analysis of diverse radiological manifestations in five confirmed cases of DCC. Patients presented with varied symptoms, including localized swelling, pain, tingling sensations, and breakthrough seizures. High-resolution ultrasonography (USG) revealed typical cystic lesions, with or without scolex, surrounded by inflammatory changes or calcifications, while computed tomography (CT) and magnetic resonance imaging (MRI) provided additional confirmation, especially in neurocysticercosis cases. Imaging patterns included myo-cysticercosis, soft tissue cysticercosis, orbital involvement, and nodular-calcified neurocysticercosis. Recognition of these radiologic patterns is vital for prompt diagnosis and appropriate management, particularly in endemic regions. USG proved invaluable as a non-invasive, cost-effective, first-line tool for diagnosing soft tissue involvement, while CT and MRI were essential in evaluating central nervous system (CNS) lesions and disease staging.

## Introduction

Cysticercosis is a parasitic infection caused by the larval stage (*Cysticercus cellulosae*) of the pork tapeworm *Taenia solium*. Humans become accidental intermediate hosts through ingestion of eggs via the fecal-oral route, leading to the development of cysticerci in various tissues. While neurocysticercosis is the most prevalent and well-documented form, disseminated cysticercosis (DCC) is a rare but significant manifestation, characterized by widespread cysticerci affecting muscles, subcutaneous tissues, eyes, and the central nervous system (CNS) [1].

Though fewer than 50 cases of DCC have been reported globally, the majority originate from endemic regions such as India, Latin America, and Southeast Asia [2]. Clinical presentations can be highly variable, including muscle pain, swelling, seizures, ocular symptoms, or even asymptomatic calcifications. These diverse manifestations often mimic neoplastic, inflammatory, or infectious conditions, making accurate diagnosis a challenge [3].

Radiology plays a pivotal role in detecting, characterizing, and staging cysticercosis. Ultrasonography (USG) is especially valuable for soft tissue involvement, revealing cysts with or without a visible scolex, surrounding edema, or abscess formation. Computed tomography (CT) and magnetic resonance imaging (MRI) are essential for identifying neurocysticercosis and calcified lesions, with hallmark imaging features such as ring-enhancing lesions and gradient recalled echo (GRE) blooming in chronic stages [4].

This case report aims to highlight the radiological spectrum of DCC through case-based analysis, with a particular focus on the utility of ultrasound and cross-sectional imaging. By improving recognition of these imaging patterns, clinicians and radiologists can make timely diagnoses and avoid unnecessary interventions.

## Case presentation

Case 1: Myo-cysticercosis - pseudotumor type involving the triceps muscle

A 26-year-old male presented to the Radiology Department with complaints of progressive pain and swelling over the posterior aspect of the left arm for the past one year. The symptoms were insidious in onset and gradually progressive. There was no history of trauma, fever, or systemic signs of infection. Clinical examination revealed a localized, mildly tender swelling in the posterolateral aspect of the arm, without signs of erythema or fluctuation.

**Figure 1:**
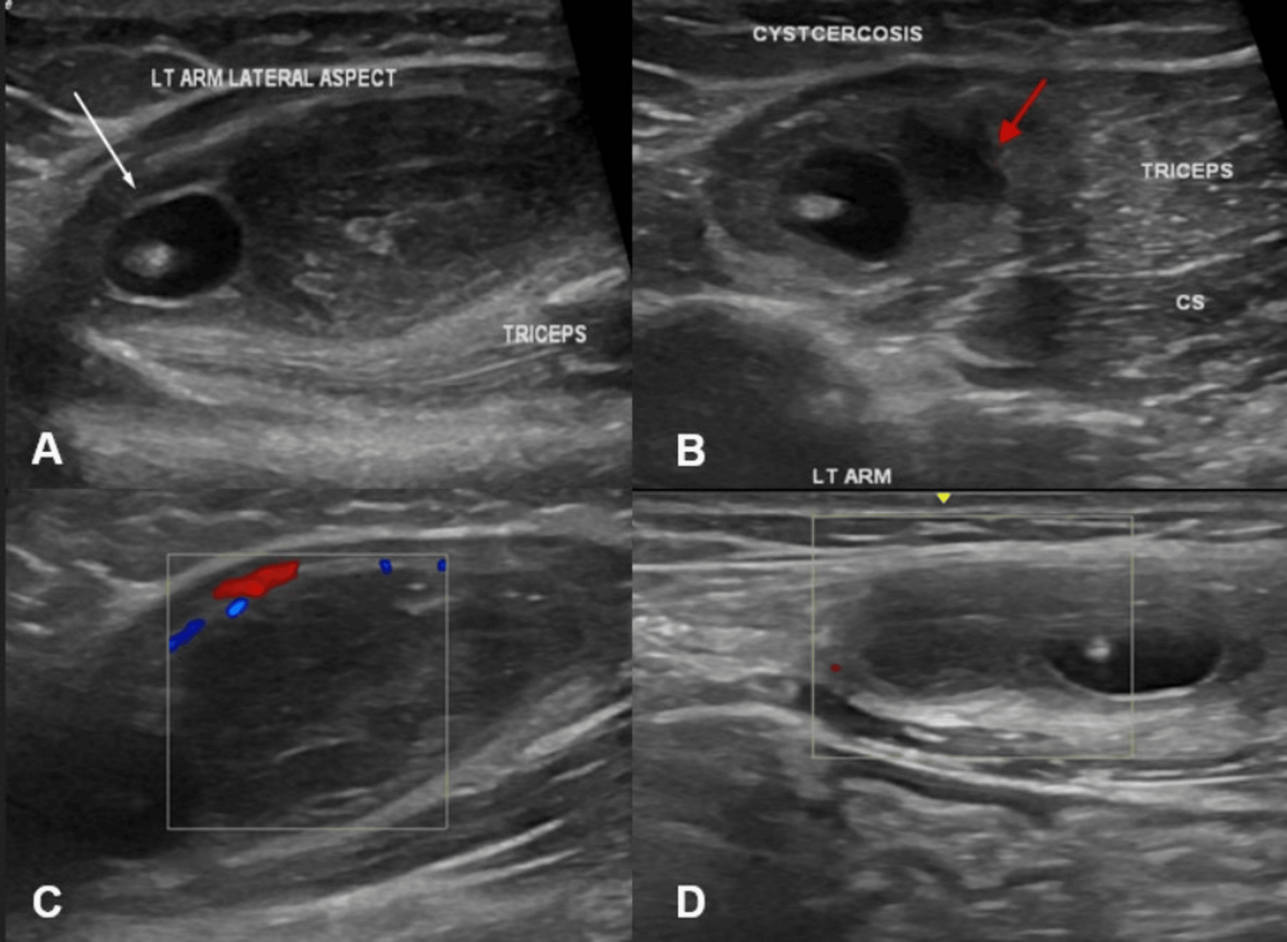
Ultrasound of left arm showing intramuscular abscess in triceps muscle, along the direction of longitudinal fibers of the muscle Ultrasound images of the left arm in longitudinal section (A) and cross-section (B) showing a well-defined cystic lesion with an eccentrically placed echogenic nodule (indicated by white arrow) and surrounding intramuscular abscess (indicated by red arrow) seen in the triceps muscles. Color Doppler images in longitudinal section (C) and cross-section (D) showing mildly increased peripheral vascularity around the hypoechoic area - likely surrounding inflammatory changes.

USG of the left arm was performed, revealing a well-circumscribed, anechoic cystic lesion within the triceps muscle. A distinct, eccentrically located echogenic nodule was visualized within the cyst, consistent with the scolex of *C. cellulosae* (Figure 1A). Surrounding the lesion, an irregular hypoechoic area was noted (Figure 1B), with mildly increased peripheral vascularity on Doppler imaging (Figures 1C-1D), suggestive of inflammatory changes and early abscess formation. The lesion was elongated and seen along the direction of the muscle fibers, which is a classic sign seen in muscular cysticercosis.

Based on the USG findings, a diagnosis of myo-cysticercosis, pseudotumor (abscess) type, was made. This subtype results from chronic inflammation and immune response to leakage of cyst fluid, leading to abscess formation around the larval cyst. The patient was advised anti-helminthic therapy (albendazole), in combination with corticosteroids, to control inflammation and prevent paradoxical worsening due to larval death.

Case 2: Soft tissue cysticercosis - muscular and subcutaneous involvement

A 42-year-old female presented with complaints of a tingling/crawling sensation and mild discomfort in her right arm and left leg for four months. The symptoms were not associated with significant pain or swelling, but she reported intermittent paresthesia over the affected areas. There was no fever or neurological deficit. Her primary physician referred her for soft tissue imaging.

**Figure 2:**
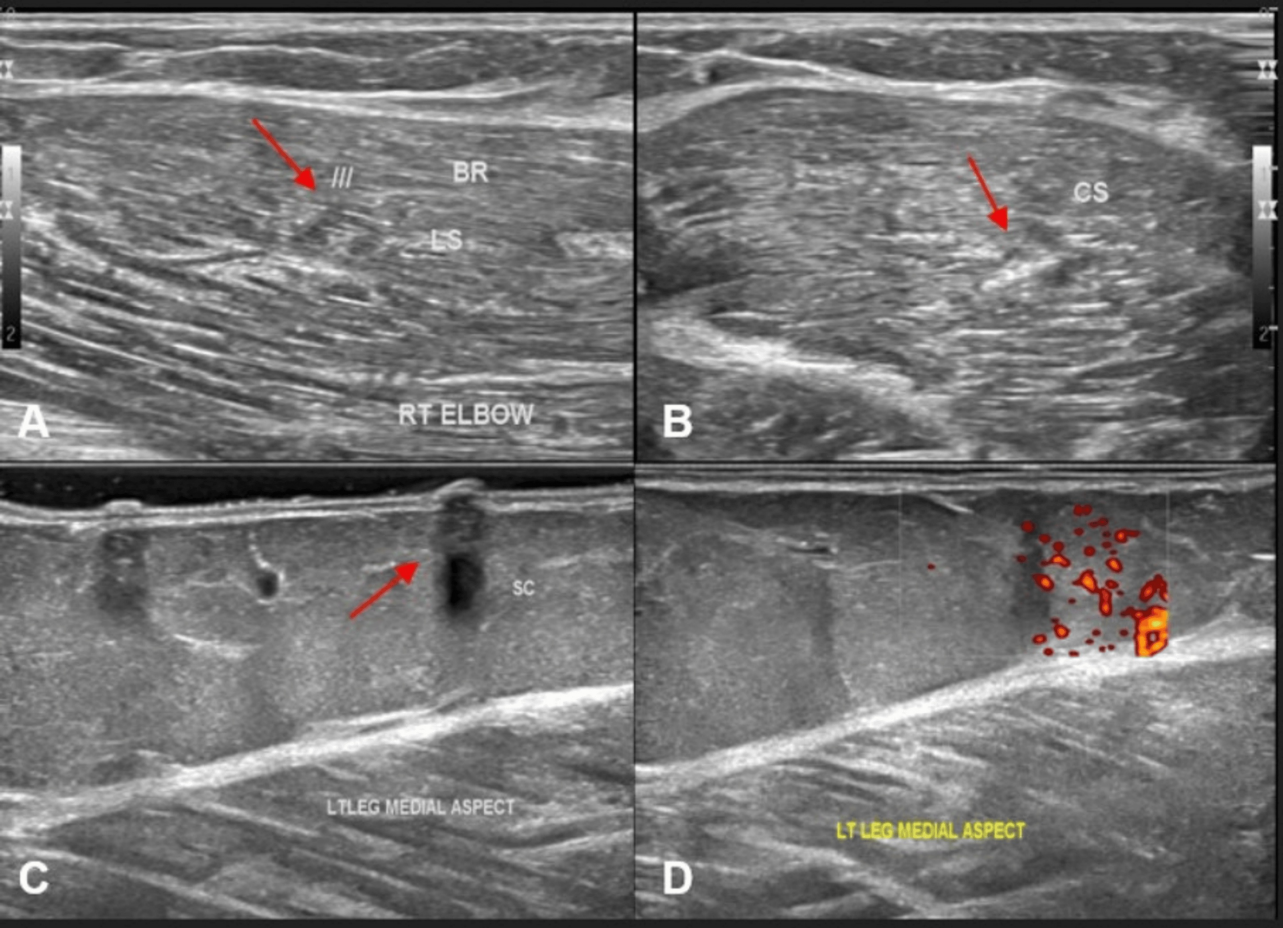
Ultrasound of right elbow (A, B) and left leg (C, D) showing soft tissue cysticercosis Ultrasound images of the right elbow in longitudinal section (A) and cross-section (B) showing a well-defined oval hypoechoic area (indicated by red arrows) measuring ~5 x 5 mm seen in the brachioradialis muscle - likely uncalcified cysticercosis. Ultrasound images of the left leg in longitudinal section (C) showing an irregular hypoechoic area (indicated by red arrow) measuring ~7 x 4 mm in the subcutaneous plane, with increased vascularity on power Doppler imaging (D) - likely inflammatory changes in the subcutaneous plane.

USG of the right elbow showed a well-defined, oval hypoechoic lesion measuring approximately 5 × 5 mm, located within the brachioradialis muscle (Figures 2A-2B). There was no evidence of calcification, surrounding abscess, or edema formation, indicating an early or viable cyst stage. In the left leg, an irregular hypoechoic lesion measuring approximately 7 × 4 mm, with similar characteristics, was noted in the subcutaneous tissue. Power Doppler imaging revealed increased vascularity surrounding the lesion (Figures 2C-2D), suggestive of active inflammatory changes.

The combined imaging pattern across two anatomically distant sites confirmed disseminated soft tissue cysticercosis, with muscular and subcutaneous involvement. The presence of increased vascularity and edema in the subcutaneous lesion suggested leakage of cyst contents, triggering a local immune reaction. The patient underwent a complete blood investigation, which showed eosinophilia. She was started on albendazole therapy, with oral steroids.

Case 3: Orbital and digital cysticercosis in a pediatric patient

A 13-year-old female was brought to the clinic by her parents with complaints of a painless swelling in the left upper eyelid and dorsum of the right little finger, both gradually increasing in size over the past three months. There was no associated fever, vision impairment, or signs of infection. The eyelid swelling was soft, mobile, and non-tender, with no conjunctival injection or discharge. A local soft tissue infection was suspected clinically.

**Figure 3:**
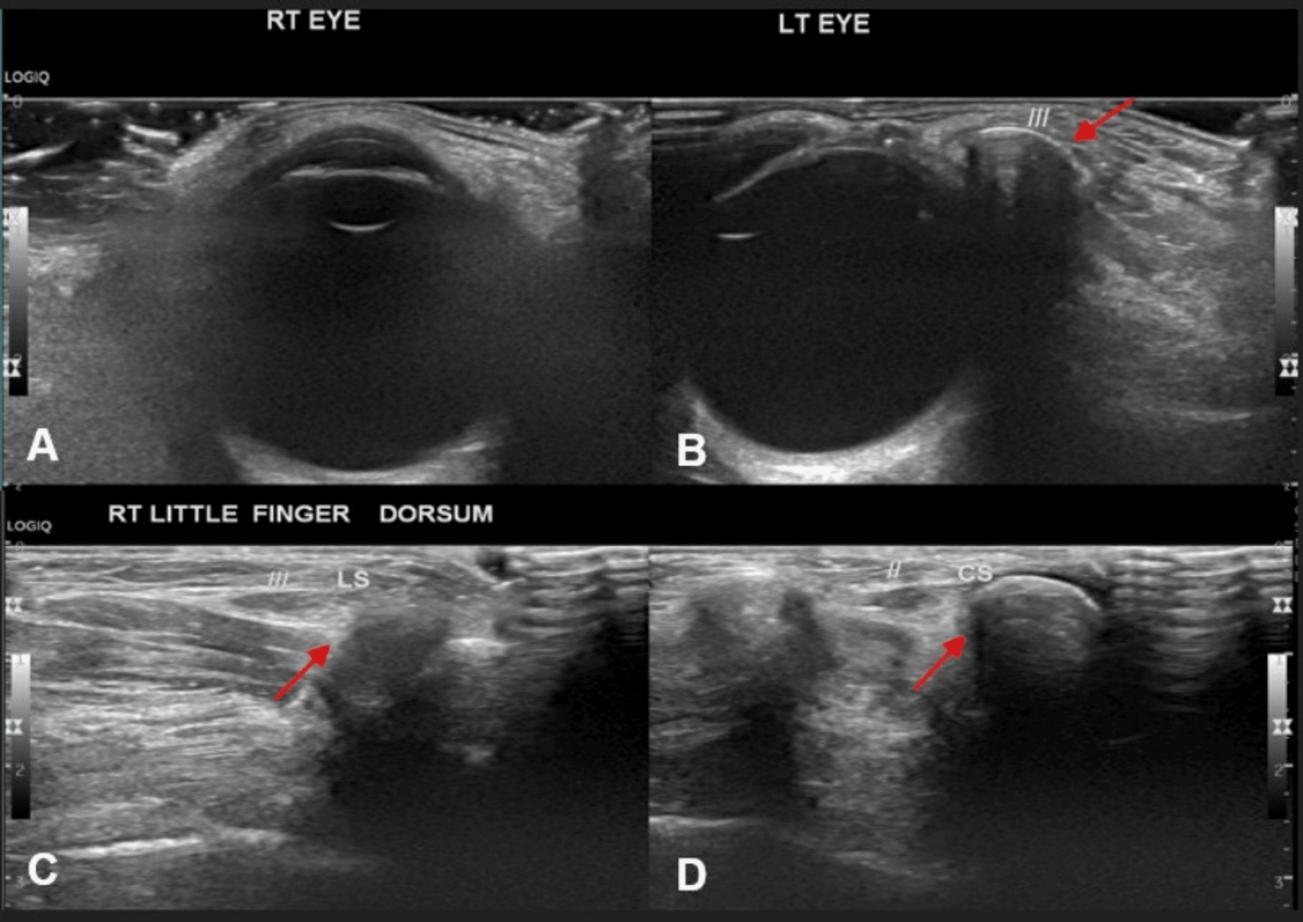
Ultrasound of the left eye and right little finger showing orbital and adnexal cysticercosis Ultrasound image of normal right eye (A) for comparison. Ultrasound image of the left eye (B) showing a well-defined oval hypoechoic area (indicated by red arrow) measuring ~10 x 6 mm seen in the subcutaneous plane of the left orbit - likely uncalcified cysticercosis. Ultrasound images of the dorsum of the right little finger in longitudinal section (C) and cross-section (D) showing a well-defined oval hypoechoic area in the muscular plane (indicated by red arrows) - likely uncalcified cysticercosis.

USG image of the normal right eye for comparison (Figure 3A) and USG of the left orbit demonstrated a well-defined, oval hypoechoic lesion measuring 10 × 6 mm, located in the subcutaneous plane of the left orbit (Figure 3B). No calcifications were seen. A well-defined hypoechoic lesion with similar characteristics was identified in the muscular plane over the dorsum of the right little finger (Figures 3C-3D), without signs of inflammation or calcification.

These findings were consistent with uncalcified orbital and muscular cysticercosis in a viable stage. Orbital involvement, though uncommon, is clinically significant due to potential complications like orbital cellulitis, proptosis, or diplopia if left untreated. In this case, early detection allowed for conservative management with albendazole and steroids. A brain MRI was also advised to rule out concurrent neurocysticercosis, as ocular involvement often coexists with CNS lesions.

Case 4: Disseminated calcified cysticercosis - incidental finding in an elderly female

**Figure 4:**
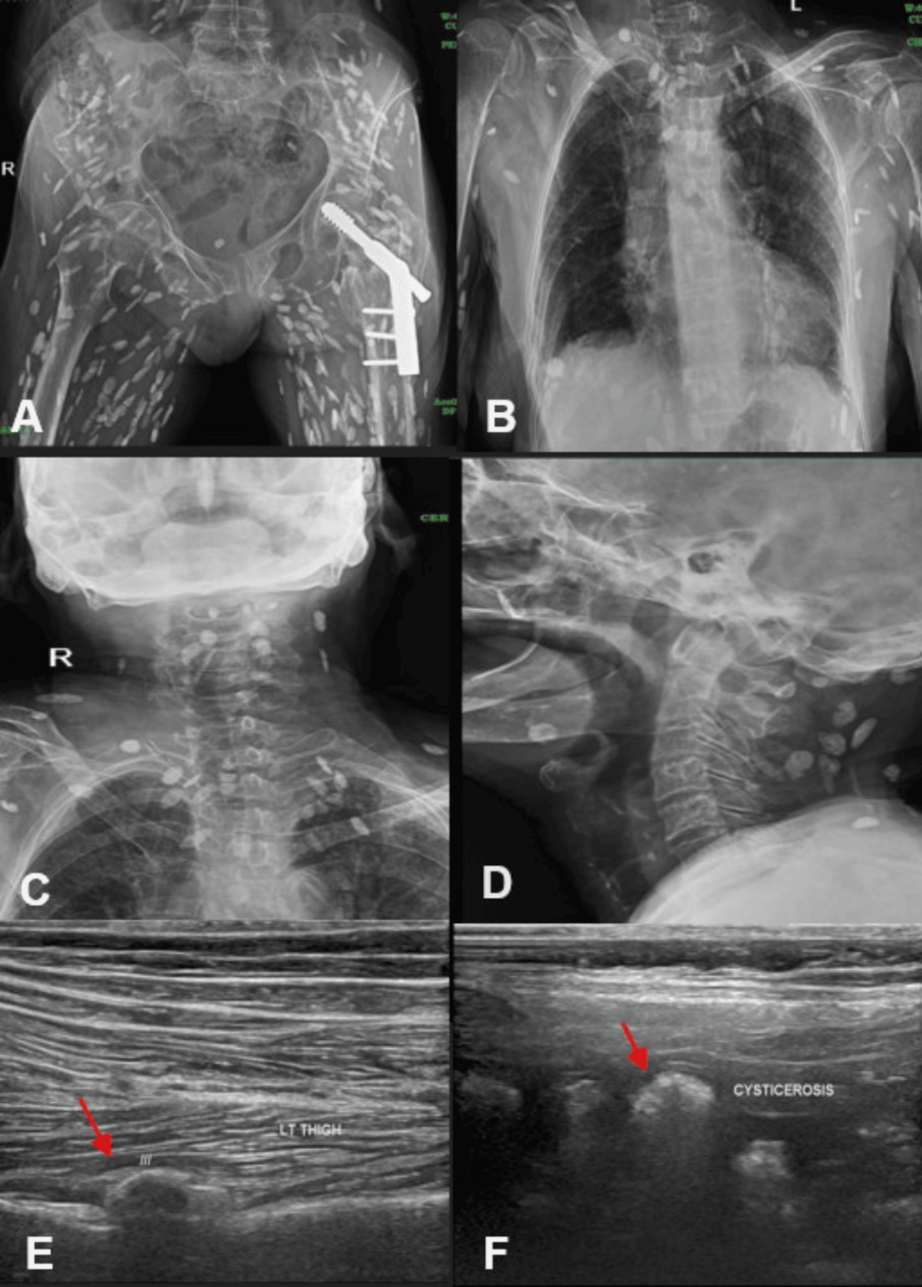
Radiographic and ultrasound images showing classic "rice grain pattern" of calcified cysticercosis Radiographic images (A-D) showing multiple typical rice grain appearances in the subcutaneous and muscular plane - suggestive of calcified cysticercosis. Ultrasound screening images of the thigh (E-F) showing multiple hyperechoic lesions with posterior acoustic shadowing (indicated by red arrows) - suggestive of calcified cysticercosis.

An 81-year-old female with a history of giddiness and fall was referred for imaging to assess potential skeletal injuries. She had no history of seizures, cognitive impairment, or systemic illness. Radiographic imaging of the pelvis and thighs revealed multiple elongated calcific radio-opacities giving the classic “rice grain appearance” (Figures 4A-4D), scattered within the muscular and subcutaneous planes. These findings were highly characteristic of calcified cysticerci in the chronic stage.

USG evaluation of the pelvis and thighs further confirmed the presence of multiple hyperechoic linear lesions with posterior acoustic shadowing (Figures 4E-4F), consistent with calcified cysticercosis. There were no surrounding hypoechoic areas, edema, or increased vascularity, indicating an absence of active inflammation or live cysts.

This case represented asymptomatic disseminated calcified cysticercosis, an incidental radiological finding. Such lesions do not typically require treatment unless associated with clinical symptoms. The patient was reassured and managed conservatively.

Case 5: Neurocysticercosis in a seizure patient - nodular calcified stage

A 51-year-old female, known to have a seizure disorder for the past two years, presented with breakthrough episodes despite adherence to antiepileptic medications. There was no history of fever, recent trauma, or focal neurological deficit. Her previous medical records lacked documentation of neuroimaging. She was referred for CT and MRI evaluation to reassess seizure etiology.

**Figure 5:**
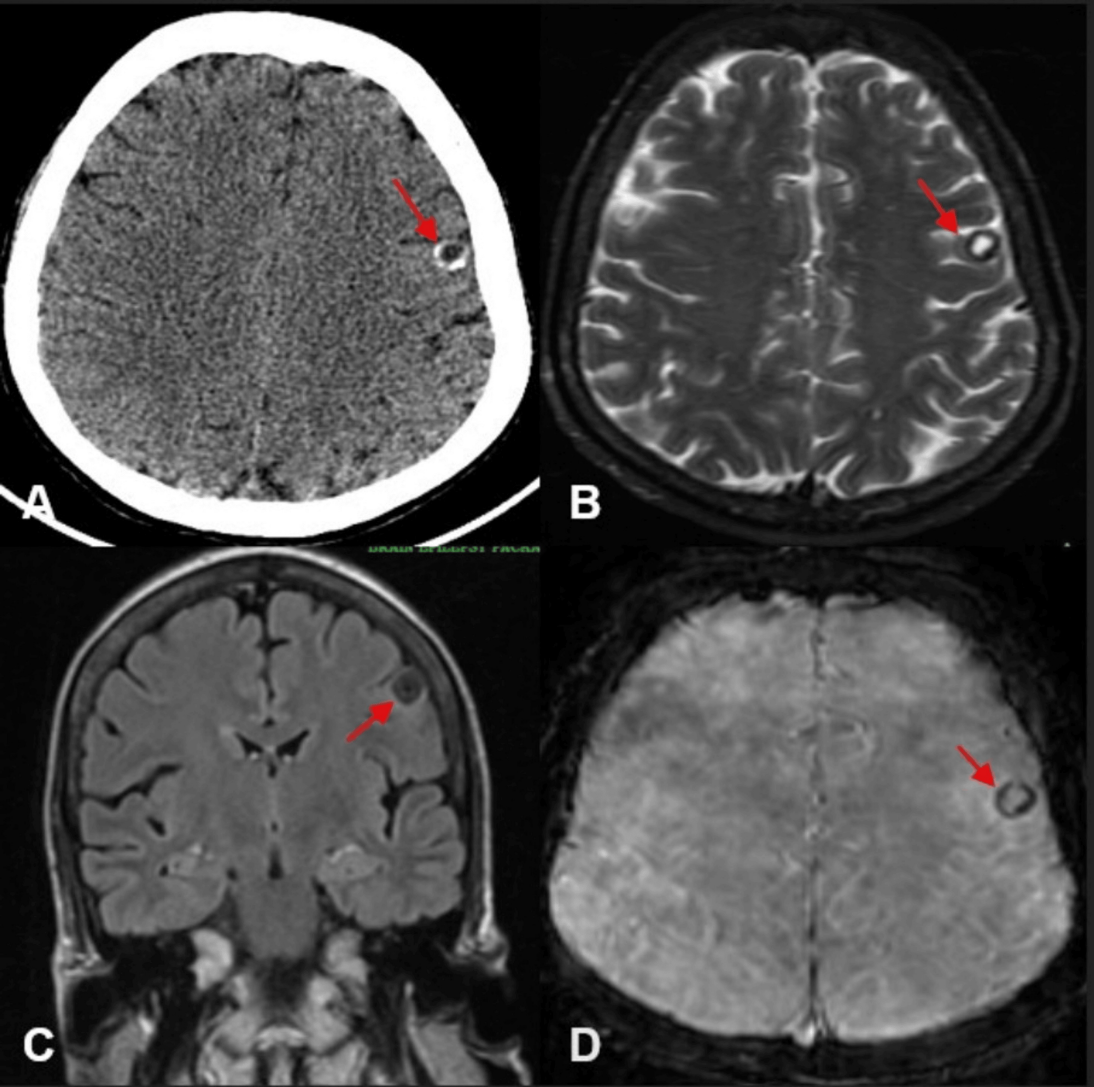
CT and MRI of brain showing neurocysticercosis with wall calcification Axial NCCT image of the brain (A) showing a well-defined hypodense (CSF density) lesion with a peripheral rim of calcification (indicated by red arrow) in the left frontal lobe. MRI brain shows (B) a T2 hyperintense lesion (indicated by a red arrow), (C) FLAIR suppression in the coronal image (indicated by a red arrow), and (D) a peripheral rim of GRE blooming (indicated by a red arrow). NCCT, Non-contrast Computed Tomography; FLAIR, Fluid-Attenuated Inversion Recovery; GRE, Gradient Recalled Echo; MRI, Magnetic Resonance Imaging

Non-contrast CT (NCCT) of the brain revealed a well-defined hypodense lesion with a rim of peripheral calcification in the left frontal lobe (Figure 5A). MRI demonstrated that the lesion was hyperintense on T2-weighted images, with complete suppression on T2 fluid-attenuated inversion recovery (FLAIR) sequences, indicating a cerebrospinal fluid (CSF)-like fluid content within the cyst (Figures 5B-5C). GRE blooming was seen along the margin, confirming the presence of calcification (Figure 5D). These findings were diagnostic of nodular-calcified neurocysticercosis.

The seizures were attributed to the edema/inflammatory changes surrounding the calcified lesion. The patient was advised to continue antiepileptic drugs and was started on a short course of steroids. She was also initiated on albendazole therapy, under corticosteroid cover, to prevent inflammatory exacerbation due to larval death

## Discussion

DCC, though rare, presents with a wide spectrum of radiological manifestations depending on the organ system involved, the immune status of the host, and the stage of the parasite. The five cases presented in this report exemplify the diversity in clinical and imaging features across muscular, subcutaneous, ocular, orbital, and CNS systems.

The findings align with other contemporary case reports. For instance, a 2021 report from Tanzania also described unusual multisystem involvement, including cardiopulmonary dissemination, detected initially via coronary CT angiography, and further supported by clinical signs such as recurrent seizures and skin nodules. This underscores the critical need for whole-body imaging in atypical presentations of seizure or systemic symptoms [5].

Case 1 in this report, involving pseudotumor-type myo-cysticercosis, correlates with documented pseudotumoral forms, particularly when the larval death induces local inflammatory responses mimicking soft-tissue tumors or abscesses. Similar muscular pseudotumor presentations were emphasized in a 2023 case report, which noted diagnostic confusion with neoplastic masses due to surrounding edema and vascularity [6].

The second and third cases, involving muscular and subcutaneous cysts in multiple sites, including ocular and digital regions, mirror recent literature highlighting the expanding anatomical range of cysticercosis beyond classical CNS involvement. Rumhumha et al. (2024) reported a South African case of DCC, initially misdiagnosed as tuberculosis, highlighting the importance of differential imaging interpretations in endemic regions [7].

Orbital and digital involvement in the pediatric patient reflects observations in China, where intravitreal and muscular cystic lesions were confirmed via MRI and biopsy, particularly in the context of viable-stage larvae, with characteristic scolex visualization [8].

The incidental findings of calcified cysticerci in Case 4 reflect a known chronic sequela, often asymptomatic, and support the approach of conservative management without antiparasitic therapy in such cases. This matches findings in a chronic obstructive pulmonary disease (COPD) patient, where incidental calcifications were seen on radiographs, again affirming the “rice grain” sign in chronic inactive cysticercosis [9].

Lastly, Case 5, involving neurocysticercosis with calcified nodular lesions and seizure breakthrough, reiterates the role of GRE sequences in detecting rim calcifications. Multiple recent reports echo the significance of MRI and CT in defining cyst stage and guiding anti-parasitic therapy in seizure patients [10].

## Conclusions

DCC remains a diagnostic challenge due to its nonspecific and protean clinical presentations. Radiological imaging plays a crucial role in its identification, classification, and management. USG remains the frontline tool for detecting soft tissue lesions, revealing characteristic features such as cysts with scolex and surrounding edema or abscess. Cross-sectional modalities like CT and MRI are indispensable for confirming neurocysticercosis, staging the disease, and identifying complications. Accurate radiological interpretation, combined with clinical correlation, enables early diagnosis, reduces unnecessary surgical interventions, and guides effective medical therapy. A multidisciplinary approach, involving radiologists, neurologists, and infectious disease specialists, is key to optimizing outcomes in these patients.
